# TrkB Receptor Signalling: Implications in Neurodegenerative, Psychiatric and Proliferative Disorders

**DOI:** 10.3390/ijms140510122

**Published:** 2013-05-13

**Authors:** Vivek K. Gupta, Yuyi You, Veer Bala Gupta, Alexander Klistorner, Stuart L. Graham

**Affiliations:** 1 Australian School of Advanced Medicine, Macquarie University, F10A, 2 Technology Place, North Ryde, Sydney, NSW 2109, Australia; E-Mails: yuyi.you@students.mq.edu.au (Y.Y.); sasha.klistorner@sydney.edu.au (A.K.); stuart.graham@mq.edu.au (S.L.G.); 2 Centre of Excellence for Alzheimer’s Disease Research & Care, School of Medical Sciences, Edith Cowan University, Joondalup, WA 6027, Australia; E-Mail: v.gupta@ecu.edu.au; 3 Save Sight Institute, Sydney University, Sydney, NSW 2000, Australia

**Keywords:** neurotrophins, neurodegenerative disorders, psychiatric disorders, cancer, retina, glaucoma, TrkB receptor, BDNF, Shp2 phosphatase

## Abstract

The Trk family of receptors play a wide variety of roles in physiological and disease processes in both neuronal and non-neuronal tissues. Amongst these the TrkB receptor in particular has attracted major attention due to its critical role in signalling for brain derived neurotrophic factor (BDNF), neurotrophin-3 (NT3) and neurotrophin-4 (NT4). TrkB signalling is indispensable for the survival, development and synaptic plasticity of several subtypes of neurons in the nervous system. Substantial evidence has emerged over the last decade about the involvement of aberrant TrkB signalling and its compromise in various neuropsychiatric and degenerative conditions. Unusual changes in TrkB signalling pathway have also been observed and implicated in a range of cancers. Variations in TrkB pathway have been observed in obesity and hyperphagia related disorders as well. Both BDNF and TrkB have been shown to play critical roles in the survival of retinal ganglion cells in the retina. The ability to specifically modulate TrkB signalling can be critical in various pathological scenarios associated with this pathway. In this review, we discuss the mechanisms underlying TrkB signalling, disease implications and explore plausible ameliorative or preventive approaches.

## 1. Introduction

The Trk family of receptors includes TrkA, TrkB and TrkC which are instrumental in carrying out the cellular effects of neurotrophins. P75NTR, a member of the tumour necrosis receptor superfamily exhibits some structural similarities to Trk family of receptors and interacts with members of the Trk family including TrkB receptor and influences its signalling. There is a cross talk between various members of the Trk family and p75NTR by formation of the chimeric heteromeric complexes [1]. TrkB (EC 2.7.10.1) (*NTRK2*) molecule, in particular, has attracted significant attention in recent years because of its involvement in several important biological processes. TrkB acts as a receptor for brain-derived neurotrophic factor (BDNF) and neurotrophin-4 (NT4) ligands. Neurotrophin-3 (NT3) in addition to binding and activating TrkC, can also bind TrkB with reduced affinity and plays a role in regulating neuronal survival [1]. The tyrosine kinase receptors are activated upon binding to the cognate neurotrophin ligand and undergo dimerization with the unliganded monomeric form believed to be in equilibrium with the phosphorylated dimeric state [2]. This dynamic equilibrium between monomeric and dimeric forms may be important to regulate downstream intracellular biochemical actions of neurotrophins and their receptors. TrkB is a single pass type 1 membrane protein and may be incorporated in endosomes upon ligand binding [3,4]. This receptor contains a protein kinase domain, two leucine rich repeats and two Ig-like C2 set domains. TrkB is expressed in both the central (CNS) and peripheral nervous systems (PNS). In the CNS, a high TrkB expression is observed in cerebral cortex, hippocampus, thalamus, choroid plexus, and granular layer of the cerebellum, brain stem, retina and the spinal cord [5]. In the PNS, it is expressed in the cranial ganglia, vestibular system, sub-maxillary glands and the dorsal root ganglia [5]. TrkB is widely expressed in the fetal brain. TrkB expression is also detected in a variety of other tissues like skeletal muscles, kidneys and pancreas. Truncated forms of TrkB lacking the tyrosine kinase domain are known to be distributed in heart, ovary and spleen tissues [6]. TrkB expression has also been observed in the Meissner corpuscles [7].

## 2. Biological Functions of TrkB Signalling

**Figure 1:**
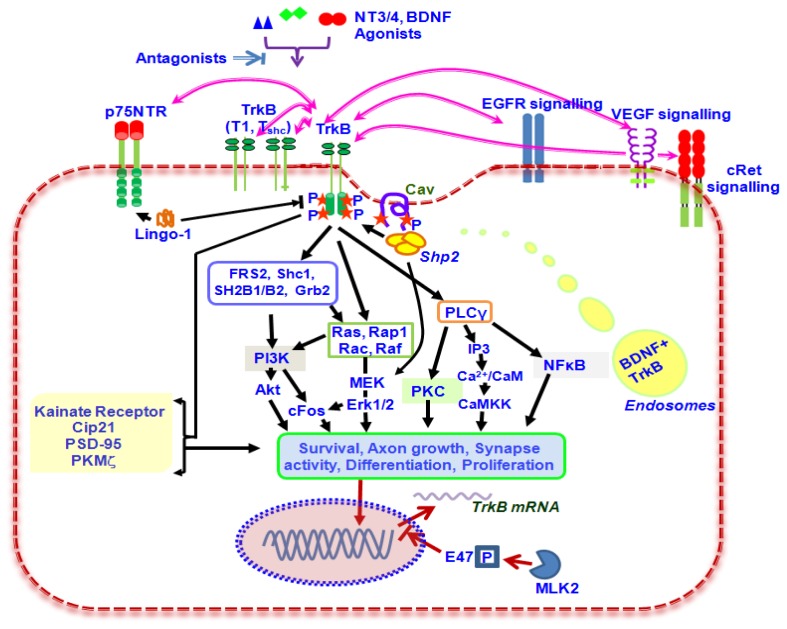
Schematic representation of the BDNF**/**TrkB receptor signalling axis and its downstream cellular effects. The binding of neurotrophic factors leads to auto-phosphorylation of the receptor intracellular domain. Cross-talk with other receptors and its regulation is shown. The internalization and transport of BDNF/TrkB in cells through endosomes is represented diagrammatically. P, Phosphorylation; Cav, Caveolin; IP3, Inositol tri-phosphate; CaM, Calmodulin.

Trk receptors possess an intracellular tyrosine kinase domain which upon phosphorylation recruits signalling intermediates and initiate intracellular signalling cascades (Figure 1). The tyrosine phosphorylation is triggered by the binding of neurotrophins leading to recruitment of pleckstrin homology (PH) and SH2 domain containing proteins such as FRS2, Shc, SH2B and SH2B2 that regulate distinct overlapping signalling cascades [8,9]. TrkB regulates growth and survival of the cells by controlling the Ras-PI3K-Akt signalling cascade. There is sufficient evidence that in the neurons, TrkB activates the GRB2-Ras-MAPK-Erk signalling and regulates the neuronal differentiation including neurite development [10,11]. TrkB activation regulates synaptic plasticity by promoting the phospholipase Cγ (PLCγ) mediated pathways through downstream protein kinase C signalling [12]. There is a cross-talk of the TrkB receptor signalling with small G proteins as well, including Ras and Rap-1, and it also interacts with Tiam1 protein leading to Rac activation [13]. TrkB has also been found to interact with p62 and ankyrin rich membrane spanning (ARMS) proteins in the cells leading to modulation of the kinetics of MAPK/Erk activation [14,15]. TrkB is involved in the transcription regulation through its effects on cell cycle inhibitor p21 (Cip21), involving helix-loop-helix proteins [16]. The transcription factor E47 upon phosphorylation by Mixed lineage kinase (MLK2) leads to inhibition of the E47 induced TrkB transcription in the neuronal cells [17]. TrkB also plays a role in neurotrophin-dependent calcium signalling in the glial cells [18–21] (Figure 1).

The pan-neurotrophin receptor, p75NTR promotes distinct signalling pathways in the cells that in most cases oppose but sometimes coordinate with TrkB promoted pathways (Figure 1). Another way of P75NTR modulating TrkB actions is through its capability to influence the receptor conformation thereby altering TrkB specificity and affinity with neurotrophins under normal and pathological conditions [12].

Various isoforms of Caveolin protein have been shown to interact with TrkB and p75NTR. Caveolar rafts in the cell membrane may further provide a platform for the interaction of these two receptors [22]. BDNF binding in fact induces a mobilisation of the TrkB.FL into the cholesterol rich lipid raft regions of neuronal plasma membrane [23]. The tyrosine phosphatase PTPN11 (Shp2) is found to play an important regulatory role in neurotrophin signalling [24]. This can be compared to the regulation of insulin receptor signalling by protein tyrosine phosphates 1-B activity in photoreceptors [25,26]. Cellular Ca^2+^ influx leads to loss of BDNF induced activation of Ras and its downstream effectors MAPK/Erk and Akt. This is the result of enhanced association of Shp2 with TrkB receptor, resulting in its deactivation. *Shp2* deletion in neuronal cultures reversed TrkB inhibition and promoted neuronal survival [27,28]. In RGCs, Shp2 binds to the TrkB receptor and plays a role in negatively regulating its activity. The inhibition of Shp2 restores TrkB activity in cells exposed to excitotoxic and oxidative stress. Caveolin-1 and 3 isoforms participate in this enhanced TrkB-Shp2 interaction illustrating a molecular basis of Shp2 mediated TrkB deactivation which leads to RGC degeneration in glaucoma [29,30]. Glucocorticoids have been shown to suppress Shp2-TrkB interaction thereby inhibiting the BDNF stimulated MAPK/Erk pathway [31] (Figure 1).

The vital role of TrkB in physiological functions is evident from the fact that TrkB^−/−^ mice die within few days after birth and display serious abnormalities in their nervous system. These mice exhibit loss of motor neurons, small dorsal root ganglion, small facial motor nucleus as well as a small trigeminal ganglion [32]. A reduced cell density is observed in cochlear ganglia [33]. TrkB pathway is vital in neural development and maintenance through differentiation and neurogenesis. Its disruption leads to unusual Purkinje cell dendrite morphology, atypical cerebellum development and diminutive geniculate and nodose ganglia [34]. It plays a role in synaptic plasticity and function [12,35]. BDNF/TrkB signalling is important in modulating presynaptic kainate receptor activity in the developing brain and in building up new synaptic connections [36]. It has a positive effect on learning and memory by regulating both short term synaptic function and hippocampal long-term potentiation [9]. TrkB signalling is indicated in both the production and maintenance of pain caused by nerve or tissue injury [37]. Owing to the fact that TrkB is well expressed in Meissner corpuscles, TrkB^−/−^ mice exhibit diminished response to tactile stimuli [32].

TrkB is a marker as well as mediator of carcinogenesis and metastasis [38]. Its expression rapidly changes in neuroblastoma cells on exposure to various treatments [39]. TrkB stimulates VEGF expression though the Phosphatidyl inositol 3-kinase (PI3K) pathway in neuroblastoma cells [40], indicating its possible role in angiogenesis. It cross-activates the Ret receptor and promotes phosphorylation of its intracellular domain. Inhibiting either TrkB or Ret leads to failure in differentiation of the neuroblastoma cells [41]. TrkB signalling is implicated in regulating protein expression and protein targeting in the neurons. TrkB receptor also cross-talks with EGFR and enhanced EGFR signalling was observed in ovarian cancer cells in response to BDNF binding leading to cell survival signalling activation [42]. TrkB stimulation triggers NF-κB activity through PLCγ1 activation and enhances cell survival signalling which in turn leads to anoikis suppression [43]. An increased c-fos expression in TrkB expressing cells upon BDNF stimulation indicates an increased survival activity, which is lost upon pharmacologically inhibiting TrkB. c-fos is a proto-oncogene and a transcription factor that promotes cellular proliferation. It’s upregulation is believed to be mediated in part through activation of both MAPK and PI3K/Akt pathways by TrkB [44]. Both follicle stimulating hormone and hepatocyte growth factor act on the cells by promoting the expression of TrkB receptor [43,45].

In the retina, TrkB is well expressed early during the development and is important in the formation of inner retinal network [46]. TrkB activation leads to an enhanced PI3K/Akt and Erk1/2 signalling in the retinal ganglion cells (RGCs) and Erk1/2 in particular, appears to be responsible for promoting the survival of RGCs [47]. TrkB mediates BDNF internalization and its retrograde transport from the retina to isthmo-optic nucleus [48]. TrkB signalling programmes the differentiation of retinal progenitor cells to the photoreceptors and both BDNF and TrkB have been shown to be involved in mediating retinal neuroprotection [49–51]. Rod photoreceptors are conspicuous in that these lack TrkB, but TrkB signalling may be indirectly supported through the Müller cells and retinal pigment epithelium in the retina. TrkB signalling is also important in the conversion of Müller glia to photoreceptors during development [52,53]. A truncated isoform of TrkB receptor, TrkB.T1 in Muller cells is implicated in BDNF-mediated photoreceptor protection against light damage [54]. TrkB along with its ligands is observed to localise to endosomes along with downstream signalling effectors, such as Shc and PLC-γ1. Cellular transport of these endosomes makes it possible for TrkB to travel through the cell body, axons and nucleus [12]. This may be the mechanism of BDNF/TrkB transport along the axons of the optic nerve. TrkB promotes PSD-95 localisation to synapses in the visual cortex along with protein kinase M zeta after eye-opening during development indicating its role in the regulation of synaptic plasticity along visual pathway and implications in developmental disorders of brain [55] (Figure 1).

## 3. Role of TrkB Splice Variants

The *TrkB* locus is in chromosome 9 in humans and is mainly expressed in three splice variants. TrkB full length (TrkB F.L.) possesses an intracellular tyrosine kinase domain and rapidly transmits the effects of neurotrophin binding to downstream network. Alternative splicing of the primary transcript produces truncated TrkB isoforms (T1 and T-shc in humans; T1 and T2 in rat) that lack the intracellular tyrosine kinase domain and cannot elicit rapid intracellular signalling [3,56]. TrkB-T-TK (Uniprot: Q16620-6) is another isoform in which *C*-terminal part (87 residues) is missing while a large part of the amino (156 residues) and carboxy-terminal (345 residues) are missing in TrkB-N-T1 (Uniprot: Q16620-7), with both isoforms exhibiting some sequence variations from the canonical TrkB sequence (Uniprot: Q16620). TrkB-T1 is predominantly expressed in the brain but also detected in heart, kidney and pancreas. Isoform TrkB-T-Shc is mainly expressed in the brain [3,57]. T1 can behave as a dominant negative isoform by competing with neurotrophins and form heterodimers with the TrkB full-length (TrkB.FL) [58]. Increased expression of T1 in injury or seizure is believed to sequester and limit the availability of BDNF, thereby preventing axonal regeneration at the site of injury [59]. The dominant negative behaviour is just one of the several functions of TrkB.T1. TrkB.T1 may additionally, sequester and translocate BDNF, induce filopodia and neurite outgrowth, stimulate intracellular signalling, regulate Rho GTPase signalling, and modify cytoskeletal structures [60]. TrkB regulates the dendritic development pattern and may play critical roles during development; TrkB.FL plays a more prominent role in increasing proximal dendritic branching whereas truncated TrkB is involved in the elongation of distal dendrites [61]. BDNF application produces rapid signals in neurons through TrkB.FL and the voltage gated sodium channel [62,63] while astrocytes predominately express TrkB.T1 and respond to BDNF by releasing calcium from the intracellular stores [64].

## 4. Involvement of TrkB Signalling in Disease Processes

**Figure 2:**
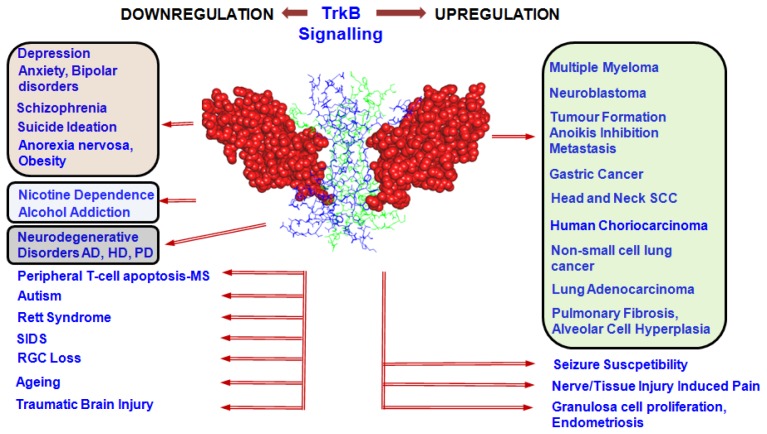
TrkB signalling alterations in various disease conditions. An increase (**right**) or decrease (**left**) of TrkB expression or its signalling may lead to a range of pathological conditions indicating the significance of the maintenance of a fine equilibrium of the TrkB signalling pathway. TrkB (space-filled)-BDNF (wire-frame) dimeric complex. AD, Alzheimer’s Disease; HD, Huntington’s Disease; PD, Parkinson’s Disease; SIDS, Sudden Infant Death Syndrome; RGC, Retinal Ganglion Cell; MS, Multiple Sclerosis; SCC, Squamous Cell Carcinoma.

An increasing number of studies have suggested the involvement of BDNF/TrkB in various pathological conditions. BDNF/TrkB dysregulation leads to cellular proliferative changes as well as degenerative and behavioural changes in the brain (Figure 2).

### 4.1. TrkB in Proliferative Disorders

TrkB is indicated to play a role in various types of malignancies including those of neuroendocrinal origin [65]. A high expression of TrkB is associated with neuroblastomas and co-operativity has been observed between TrkB and c-Met receptors in promoting the neuroblastoma invasiveness [66,67]. Differences in the gene expression pattern of TrkB lead to differential phenotypes of TrkB expressing neuroblastomas [68]. TrkB activity in general, plays an important role in tumor formation and metastasis [69]. TrkB is a strong suppressor of anoikis, which makes it an important factor in metastasis [70]. Several naturally occurring mutations in TrkB have been identified in lung adenocarcinoma patients [71]. BDNF/TrkB signalling promotes tumour cell survival in multiple myeloma by activating MAPK and PI3K/Akt pathways [72]. TrkB has also emerged as an important prognostic marker in gastric cancer [73]. TrkB forms a significant component of head and neck squamous cell carcinoma and constitutes a target for potential therapy [74]. Its activation is implicated in non-small cell lung cancer and promotes proliferation of adenocarcinomic human alveolar basal epithelial cells and metastasis via the Pyk2/ERK pathway [75]. Abnormal TrkB regulation leads to pathological lesions observed in pulmonary fibrosis, fibroblast proliferation and alveolar cell hyperplasia [76].

### 4.2. TrkB in Psychiatric Disorders

Molecular events associated with TrkB may play a role in predisposition to depression and other psychiatric disorders as well as drug abuse. *TrkB* may in particular play an important role in the susceptibility to geriatric depression [77]. TrkB levels in the serum of clinically depressed patients are reported to be higher compared to that of the normal individuals [78]. Several *TrkB* mutations have been linked to the progression of anxiety disorders [79]. Reduction of TrkB-mediated signalling may play a role in the pathophysiology of schizophrenia and bipolar disorder [80]. Schizophrenia and other psychological disorders have been linked with sharp decline in TrkB.FL transcripts in the hippocampus [81,82]. The report that *TrkB* mRNA in the prefrontal cortex is significantly diminished in the suicide ideation patients has generated a considerable interest to study its role in the suicide behaviour [83]. *TrkB* is also indicated to determine the susceptibility towards nicotine dependence [84]. It has been shown to play a role in imparting alcohol addiction amongst Caucasian populations [85].

### 4.3. TrkB in Neurodegenerative Disorders

Loss of TrkB signalling is reported to play roles in pathogenesis of Alzheimer’s, Huntington’s and other neurodegenerative disorders. It is proposed to be a susceptibility gene contributing to Alzheimer’s disease pathology [86]. TrkB signalling helps in the maintenance of the nigrostriatal system; deficiency may contribute to the progression of Parkinson's disease [87]. Interestingly, TrkB stimulation in astrocytes drives nitric oxide production which may promote neurodegeneration in brain [88]. TrkB signalling is involved in the regulation of peripheral T cell apoptosis in multiple sclerosis [89]. Alterations in TrkB signalling contribute to an increased vulnerability to autism and impair synaptic plasticity in a mouse model of fragile-X syndrome [90,91]. TrkB expression changes indicate that it may have a role in Rett syndrome pathology [92]. Neuroprotective functions of the BDNF/TrkB axis may be reduced in the respiration nuclei in sudden infant death syndrome (SIDS) cases [93]. An increase in TrkB expression is associated with increased seizure susceptibility and epileptogenesis [94]. In glaucoma, loss of TrkB signalling leads to preferential degeneration of RGCs [95,96]. Transport obstruction in the optic nerve inhibits the retrograde delivery of TrkB from the brain to RGCs resulting in TrkB signalling deficit and eventual cell death [97,98]. Intra-ocular pressure elevation leads to increase in TrkB labelling behind the optic nerve head region and its diminution in RGCs indicating that RGCs may undergo degeneration due to its reduced availability [29,99]. Lingo-1 protein which is a negative regulator of neuronal survival and axonal regeneration and a member of Nogo66/p75NTR complex has been shown to interact with TrkB receptor in retina and negatively regulate its phosphorylation in ocular hypertension [100,101].

### 4.4. TrkB in Other Disorders

Several studies support the involvement of BDNF and TrkB in vulnerability to eating disorders. This may be due to the involvement of BDNF/TrkB signalling axis in brain in the regulation of eating behaviour and attitudes or perception towards body weight or shape. *TrkB* has been implicated in promoting genetic susceptibility to anorexia nervosa, minimum body mass index and harm avoidance [102]. Defects in TrkB are also a cause of obesity hyperphagia and developmental delay (OHPDD) disorder in patients. OHPDD is a disorder characterized by early-onset obesity, hyperphagia and severe developmental delay in speech and motor function [4]. Mice heterozygous for BDNF develop obesity related to hyperphagia while BDNF administration in rats promotes weight reduction by causing loss of appetite. Mutations in *TrkB* result in impaired ligand induced phosphorylation and downstream Erk1 activation leading to obesity related disorders [4].

TrkB supports oocyte-to-granulosa cell communication by promoting signalling between JAGGED1 and NOTCH2 leading to early follicle growth [103]. Distribution of all types of TrkB receptor transcripts has been observed in oocytes and granulosa cells [104]. Alterations in placental TrkB are observed in pregnancy associated with maternal under nutrition, type-1 diabetes and disturbances of foetal growth [105]. An increased expression of TrkB in endometrium has been observed in conditions associated with endometriosis [106] (Figure 2).

## 5. Therapeutic Potential of TrkB Pathway Modulation

Molecular strategies to regulate TrkB signalling involve its activation to address neuroprotection or psychiatric issues and its suppression in cell proliferative conditions and epileptogenesis (Figure 2). The underlying hypothesis is that these disorders are induced or promoted by changes in BDNF/TrkB signalling. Correlations between alterations in TrkB signalling in Alzheimer’s disease [107,108], Huntington’s disease [109,110], Parkinson’s disease [111], Rett syndrome [112], traumatic brain injury, [113] and ageing [114] point to the therapeutic potential of TrkB agonists. A limitation of exogenous neurotrophin administration is the cross-activation of nociceptors either directly or through p75NTR and also inducing inflammation [115,116]. Inability to deliver BDNF specifically to the target cells in sufficient quantities further exacerbates the problem. BDNF is extremely unstable in blood and does not penetrate the blood-brain barrier [117,118]. It is also susceptible to proteolytic cleavage and does not have a long half life in blood [119]. These limitations have prompted the development of various agonists and antagonists of TrkB to regulate the TrkB signalling. Synthetic or natural compounds, peptide mimetics or customised antibodies which can specifically stimulate or inhibit the TrkB receptors will be of great therapeutic relevance and are being studied extensively [120,121].

BDNF mimetics have been shown to activate TrkB signalling and prevent neuronal degeneration in rodents [2]. 7,8-dihydroxyflavone is a potent TrkB agonist which promotes TrkB signalling, ameliorates the memory defects in experimental model of Alzheimer’s disease and enhances synaptic plasticity in ageing [122]. It also enhances RGC survival [121], neuromuscular transmission [123] and therapeutic efficacy in mouse model of Rett syndrome, promotes neurogenesis and exhibits antidepressant effects [124,125]. It was found to reverse memory defects in mouse model of Alzheimer’s disease, suppress BACE1 elevation and inhibit amyloid β production [126]. Drugs like memantine significantly enhance BDNF/TrkB expression and thereby promote TrkB pathways in Alzheimer’s, Huntington’s, Parkinson’s disease and glaucoma [95,96,109,111,127]. Treatment with acetylcholinesterase inhibitors such as donepezil and galantamine rapidly activates the TrkB receptor signalling in the mouse hippocampus [128]. The toxic effects of amyloid β peptide aggregation [129,130] can be averted by the upregulation of TrkB signalling [131]. Deoxygedunin is another compound which is potent and specific agonist of TrkB and has been shown to be neuroprotective [132]. Some BDNF mimetic peptides have been designed which promote the neuronal survival in a TrkB independent manner. Although the exact mechanism of these TrkB independent actions is not known, these may arise from the binding of such peptides to the BDNF binding region of other neurotrophin receptors/p75NTR leading to downstream activation of NF-κB signalling [133,134].

In the visual system, TrkB mAb therapy triggers TrkB activation, protects retina and delays RGC death in experimental glaucoma [135]. Fibroblast growth factor-2 when applied to the optic nerve after axotomy up-regulates BDNF and TrkB in RGCs by activating the Erk and Protein Kinase-A (PKA) signalling pathways [136]. TrkB gene therapy markedly promoted RGC survival after optic nerve axotomy indicating that BDNF/TrkB axis activation can be used to protect RGCs in several optic neuropathies [47]. Interestingly, repeated administration of BDNF induces inflammation, downregulates TrkB expression in RGCs and accelerates their loss and hence it is more important to develop safer BDNF mimetic molecules and other mechanism based alternative therapeutic strategies to enhance TrkB signalling [137]. Our studies demonstrated that Shp2 phosphatase regulates TrkB signalling in RGCs. Inhibiting Shp2 expression may be a novel strategy to enhance the TrkB signalling [60]. In the RGCs TrkB activates both the PI3K/Akt and Erk1/2 pathways, but Erk1/2 appears to be primarily responsible for promoting the survival of RGCs [47]. Specifically promoting the Erk1/2 may be helpful to rescue RGCs in glaucoma and other optic nerve degenerative conditions.

The findings that TrkB is important for long term survival, differentiation, and function of neurons in the hippocampus [138], and that several antidepressant treatments not only induce hippocampal neurogenesis but that blockade of hippocampal neurogenesis inhibits their antidepressant effects, suggest that TrkB agonists might open new treatment avenues for this problem [139]. *N*-acetyl serotonin activates TrkB in a circadian manner and exhibits antidepressant effects. It also enhances neurogenesis in the hippocampus [140]. Accumulative evidence suggests that antidepressant drugs such as fluoxetine, citalopram, amitryptyline, reboxetine *etc.* act by upregulating BDNF expression in the brain leading to enhanced TrkB signalling while other studies suggest that antidepressant drugs may also transactivate TrkB receptors in the brain independent of their effects on BDNF expression [141]. Lithium which has long been used for the management of bipolar disorders and mood stabilisation enhances BDNF expression and activates TrkB receptor in the cortical neurons which imparts neuroprotection against glutamate excitotoxicity [142]. Electrical stimulation of vagus nerve is used as a strategy to treat depression and this leads to sustained activation of TrkB receptor in the hippocampal region of brain [143]. Electrical stimulation accelerates BDNF/TrkB expression and promotes axonal regeneration in the motor neurons [144]. TrkB signalling is also enhanced by diet and exercise regulation. Modulation of TrkB signalling could also have a therapeutic role in management of obesity or anorexia [145].

Conversely, Trk signalling pathways can be targeted with selective inhibitors to potentially treat neuroblastomas and other tumours [146]. TrkB signalling inhibition could provide a novel therapy for human choriocarcinoma cell proliferation [147]. TrkB inhibitor K252a can potentially be used as an anoikis-sensitizing agent in nasopharyngeal carcinoma [148]. Its inhibition has been suggested as an attractive target for the treatment of chemotherapy-resistant head and neck squamous cell carcinoma [149]. A potent peptide antagonist, cyclotraxin-B reduces TrkB activation [121]. Enhancing p75NTR expression also may have a regulatory effect on TrkB actions [150]. Lingo-1 upregulation blocks TrkB functions and may be useful in the management of cancers of neuronal origins. Alternatively, blocking Lingo-1 in retina reduces RhoA GTPase and JNK activation and enhances Akt signalling in increased intra-ocular pressure animal models and thus play a neuroprotective role [151,152]. Corticosterone has been shown to negatively regulate BDNF expression in the hippocampus and lowering corticosterone levels restores BDNF/TrkB expression [153] which may find implications in neurological disorders characterised by seizures. VEGF expression is shown to be directly related to the BDNF/TrkB signalling [154,155]. Regulation of BDNF/TrkB signalling can therefore be used as a deliberate strategy to control VEGF signalling in angiogenesis and vascular proliferative disorders [156]. Similarly, VEGF has been shown to play a role in the regulation of neurotrophin signalling [157]. It will be interesting to determine how neurotrophin signalling cross-talks with angiogenic factors and how judiciously manipulating one pathway would affect the other for eventual therapeutic purposes.

## 6. Conclusions

To summarise, the TrkB pathway represents a critical and complex signalling network that is of great relevance to the understanding of pathophysiology of several proliferative, neurodegenerative and psychiatric disorders and to the potential design of next generation therapeutic interventions and combination therapies. Despite significant progress in the field of BDNF/TrkB signalling, our understanding of the central role of this network in various biochemical and physiological processes in various neuronal and non-neuronal tissues, involvement in a range of disease conditions and potential to alter specific components of this network for therapeutic applications is very limited. In this review, we have highlighted the current understanding of mechanisms regulating TrkB receptor activation and the signalling processes mediated by it. Aberrations in TrkB signalling lead to various neuropsychiatric and neurodegenerative disorders on one hand and proliferative conditions and epileptogenesis on the other. Ability to fine-tune the balance of TrkB signalling and various effectors of this complex network *in vivo* to our advantage using specific agonists and inhibitors could advance our knowledge in a range of aforementioned disease conditions. Remarkably, studying the interactions of neurotrophic factor signalling with angiogenic factors like VEGF would also open up new avenues in the understanding and control of several vascular proliferative and neurodegenerative diseases alike.

## Abbreviations

Trk: Tropomyosin Related Kinase
P75NTR: p75 Neurotrophin Receptor
SH2: Src Homology 2
FRS2: Fibroblast growth factor receptor substrate 2
Shc: Src homology 2 domain containing
SH2B: Src homology 2 domain containing adaptor protein B
SH2B2: SH2B adaptor protein 2
MAPK: Mitogen Activated Protein Kinase
VEGF: Vascular Endothelial Growth factor
EGFR: Epidermal Growth Factor Receptor
PSD-95: Postsynaptic Density Protein-95
PTPN11: Tyrosine-protein phosphatase non-receptor type 11
Shp2: SH2 domain containing Tyrosine Phosphatse 2
Pyk2: Protein tyrosine Kinase 2
Nogo: Neurite Outgrowth inhibitor
NOTCH2: Neurogenic locus notch homolog protein 2
JNK: c-Jun *N*-terminal kinase.
